# Supplementary figures and images for: Duplication of the bodily self: a perceptual illusion of dual full-body ownership and dual self-location
Source: R Soc Open Sci. 2020 Dec 9;7(12):201911. doi: 10.1098/rsos.201911 (PMC7813251; doi:10.1098/rsos.201911)

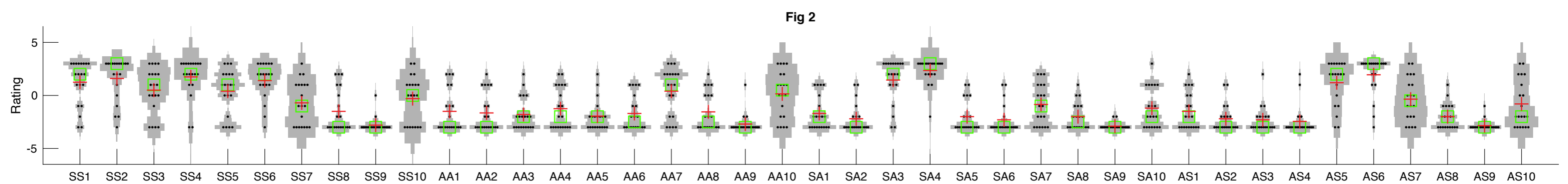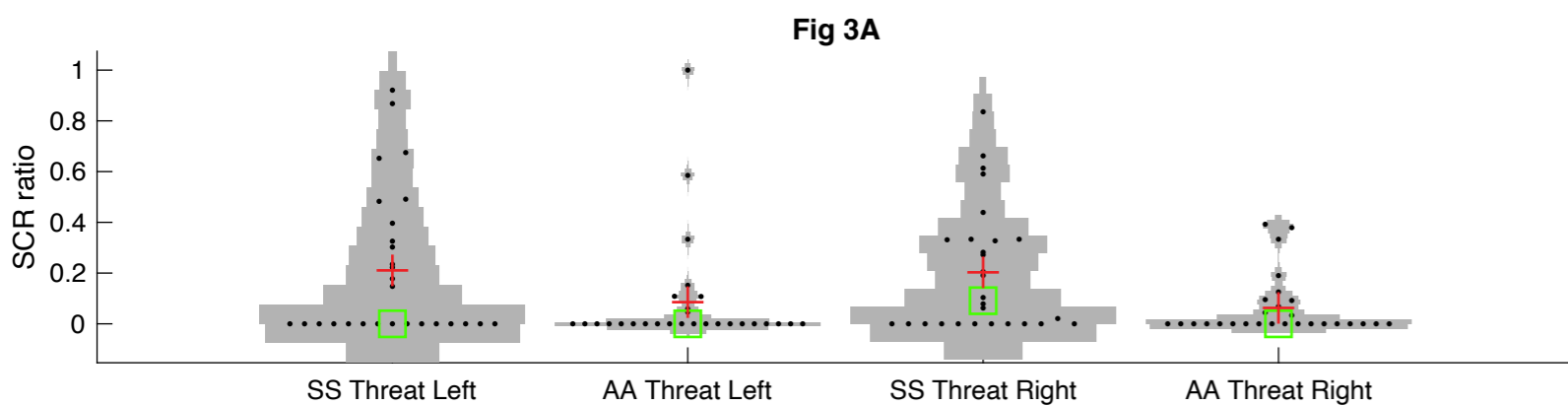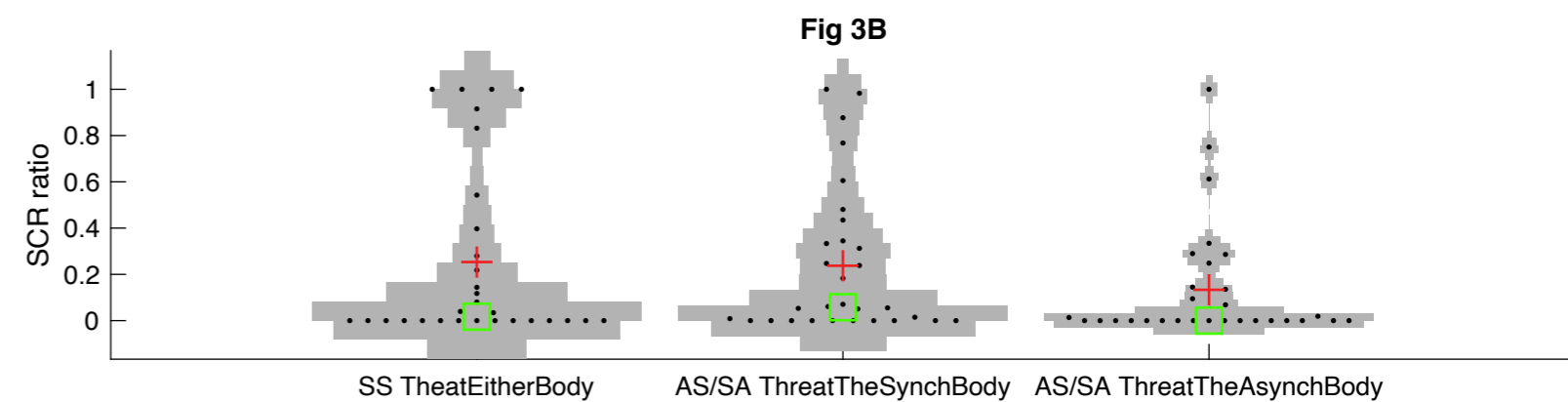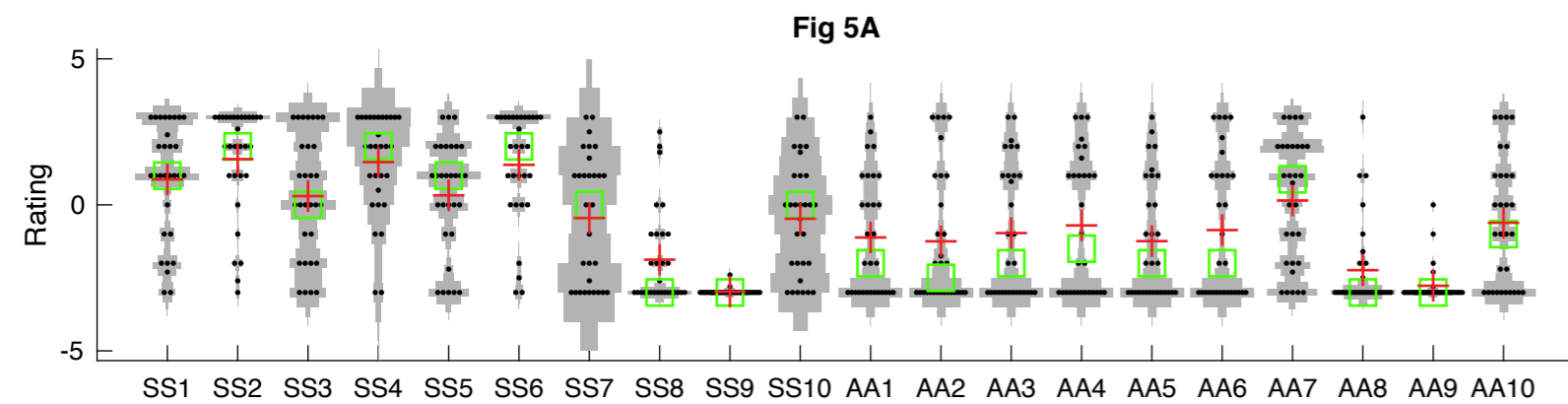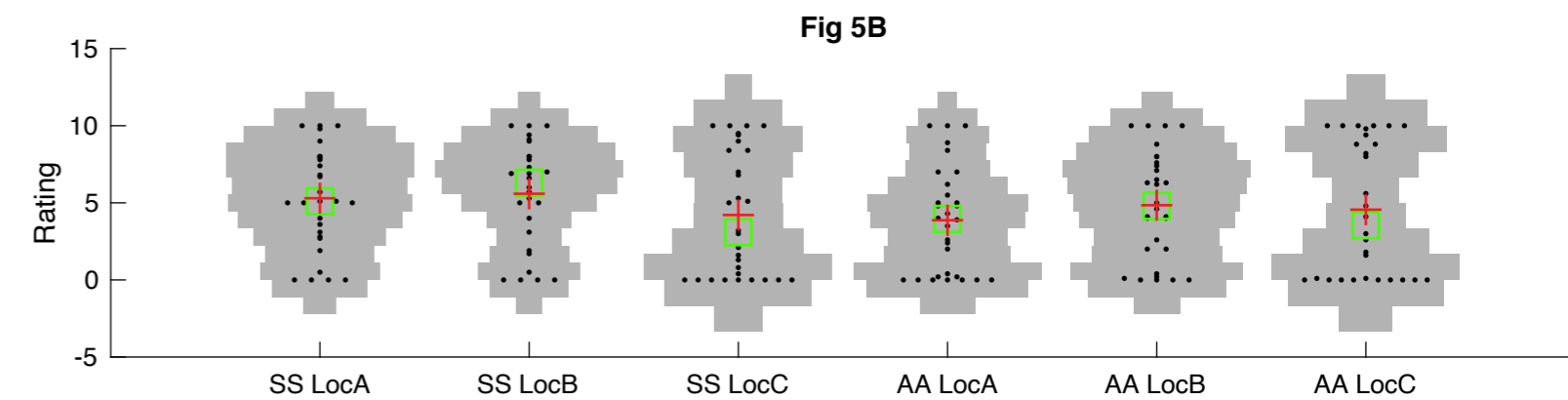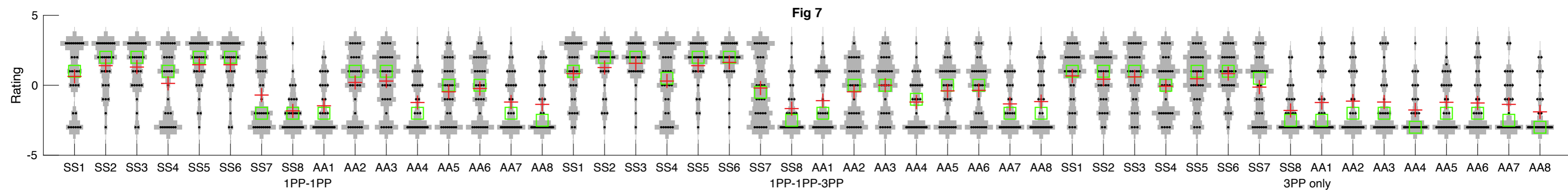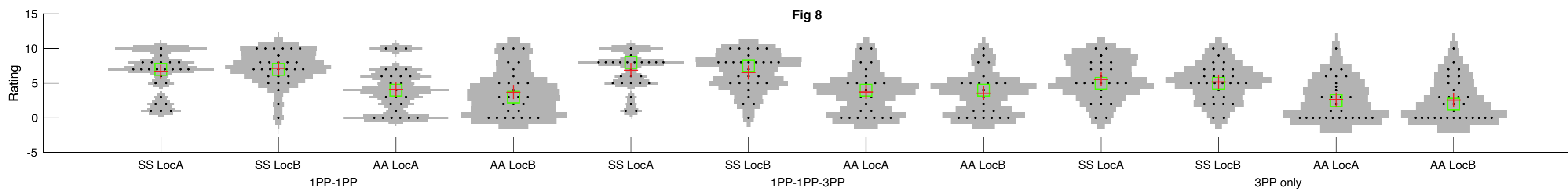

Supplement: Figure S1 [file rsos201911supp1.pdf]
